# Dystrophie maculaire de Stargardt - à propos d'un cas

**DOI:** 10.11604/pamj.2014.18.222.4871

**Published:** 2014-07-16

**Authors:** Aziz El Ouafi, Med Elmellaoui, Abdelkader Lakataoui

**Affiliations:** 1 Service d'Ophtalmologie, Hôpital Militaire Moulay Ismail, Mekhnès, Maroc

**Keywords:** Maculopathie, héréditaire, Stargardt, Maculopathy, hereditary, Stargardt

## Abstract

La maladie de Stardardt est une dystrophie maculaire héréditaire rare d'apparition précoce caractérisée par une perte progressive de la vision centrale, mais avec une vision périphérique intacte, une légère perte de la vision des couleurs, une adaptation à l'obscurité retardée, et une atrophie maculaire avec ou sans taches paramaculaires et une dégénérescence de l’épithélium pigmentaire de la rétine.

## Introduction

La maladie de Stargardt est la forme la plus fréquente de dystrophie maculaire juvénile à transmission autosomique récessive caractérisée par l'existence d'une lésion maculaire bilatérale ayant un aspect en œil de bœuf en rapport avec l'accumulation de pigment lipofiscine au niveau l’épithélium pigmentaire.

## Patient et observation

Un garçon âgé de 15 ans se plaint d'une baisse d'acuité visuelle progressive depuis 3 ans. L'acuité visuelle est chiffrée à 1/10 non améliorable en ODG. L'examen du segment antérieur aux deux yeux est sans particularités. L'examen du fond d’œil a révélé l'existence d'une lésion maculaire jaunâtre arrondie avec atrophie maculaire majeure. Le vitré est transparent, l'examen de la périphérie rétinienne est normal.

**Figure 1:**
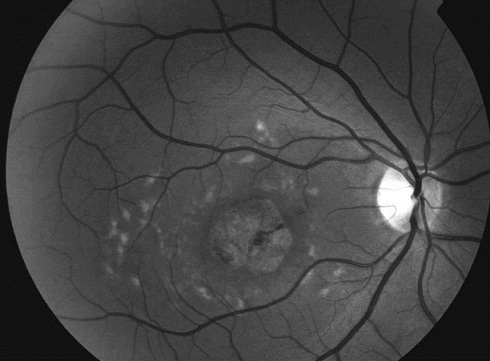
Atrophie maculaire et remaniements pigmentaires au niveau de l’œil droit

**Figure 2:**
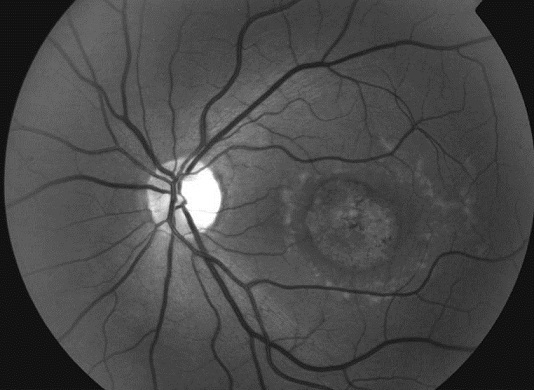
Cliché anerythre OG: aspect granulaire de la macula avec altération de l’épithélium pigmentaire

**Figure 3:**
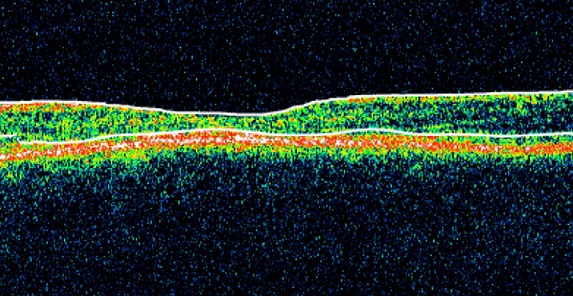
OCT de l'oeil droit: atrophie maculaire avec disparition de la ligne ellipsoïde

**Figure 4:**
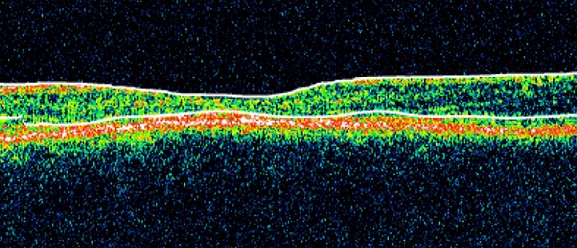
OCT de l'oeil gauche: diminution de l’épaisseur maculaire avec disparition de l'entonnoir foveolaire. Diminution de la couche des photorécepteurs

**Figure 5:**
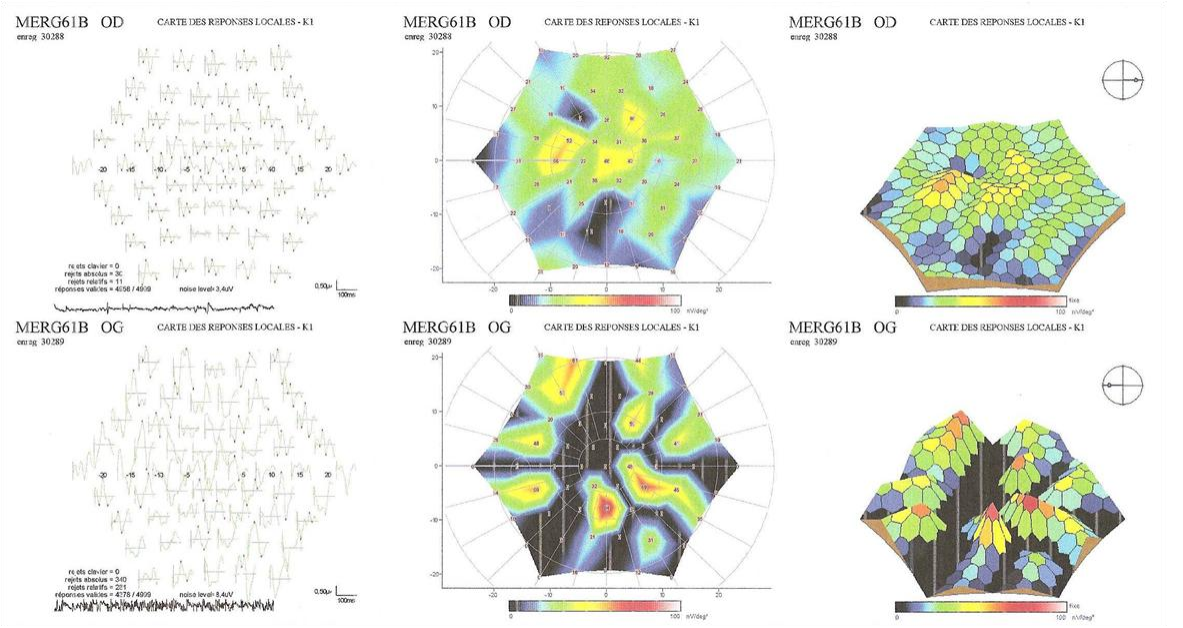
ERG full field normal

**Figure 6:**
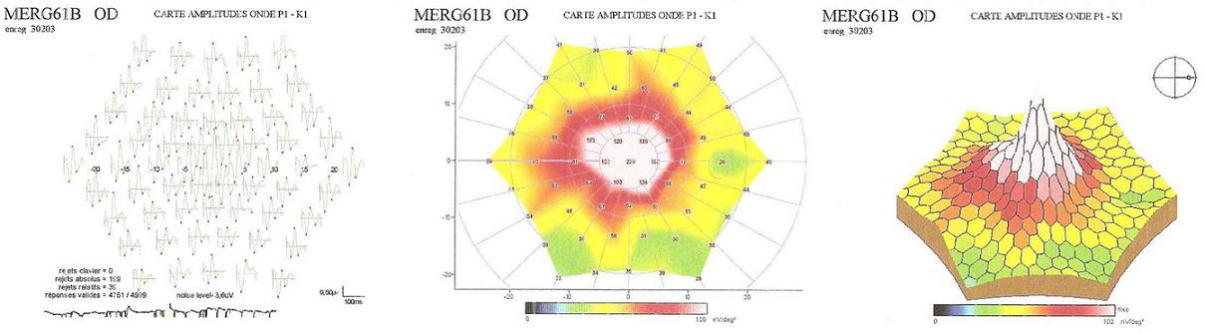
ERG multifocal normal

**Figure 7:**
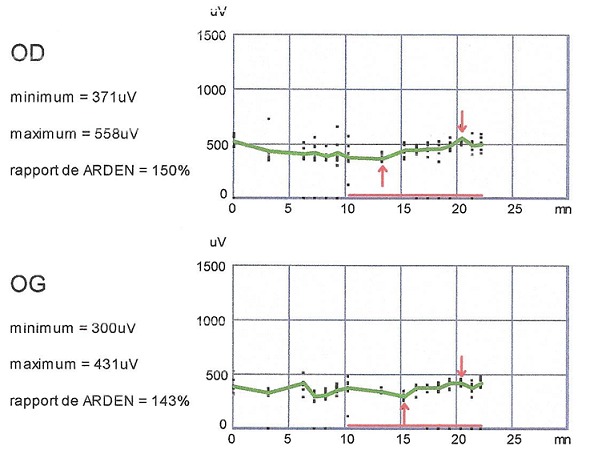
EOG normal

Les clichés en auto fluorescences décrivent un aspect granulaire des maculas avec une alternance de points hypoautofluorescents (altération de l’épithélium pigmentaire) et hyperautofl uorescents (dépôts de lipofuscine). Ces lésions sont accompagnées de taches flavimaculées, spiculées et maculaires (Figure 1, Figure 2). L'angiographie à la fluorescéine confirme la présence d'une atrophie maculaire accompagnée d'un remaniement de l’épithélium pigmentaire. Il existe un effet fenêtre comme œuf sur plat au niveau maculaire. L'OCT montre une atrophie maculaire bilatérale majeure, avec disparition de la ligne ellipsoïde (Figure 3, Figure 4). L’électrorétinogramme est normal (Figure 5, Figure 6). L’électroculogramme est normal (Figure 7). L'ensemble de ces examens paracliniques a permis de conclure au diagnostic de maladie de Stargardt de type 1.

## Discussion

La maladie de Stargardt est une maculopathie bilatérale symétrique avec dépôts pisciformes jaunes autofl uorescents [1]. Cette maladie héréditaire est à transmission autosomique récessive. Elle est liée au gène ABCA4, qui code pour une protéine permettant le transfert du tout-trans-rétinal de la lumière des disques des photorécepteurs dans leur cytoplasme. L'absence de cette protéine conduit l'accumulation de produits de dégradation du photopigment, toxique pour l’épithélium pigmentaire [2]. Cette maladie affecte surtout les enfants et les adolescents, en provoquant une baisse d'acuité visuelle progressive, accompagnée d'un scotome central et de dyschromatopsies. Le fond d’œil évolue au cours du temps et retrouve de manière précoce une altération isolée du reflet fovéolaire, oblongue, en oeil de boeuf. Il peut exister, conjointement, des taches pisciformes qui ne touchent pas la zone péripapillaire. Apparaît ensuite, tardivement, une atrophie maculaire Le diagnostic peut se faire uniquement sur les clichés autofluorescents, qui retrouvent une macula hétérogène (hypo-/hyperautofl uorescente) accompagnée de taches spiculées, hyperautofluorescentes. L'angiographie à la fl uorescéine, non nécessaire au diagnostic, montre un silence choroïdien (de Bonin), présent dans 80% des cas, ce qui permet d’éliminer certains autres diagnostics (maladie de Stargardt de transmission autosomique dominante, dystrophie des cônes, etc.).[3] L'OCT peut retrouver de manière précoce une atteinte de la ligne ellipsoïde sur la zone maculaire péri fovéale et un remaniement maculaire atrophique avec diminution de la couche des photorécepteurs. [4] L’électrorétinogramme global est classiquement normal (maladie de Stargardt de type 1) mais peut retrouver, dans certains cas, une altération des réponses scotopiques (type 2) ou photopiques (type 3), entraînant un risque d'atteinte périphérique. Le pronostic est médiocre dés que l'acuité visuelle chute au dessous de 6/10 et elle a tendance à diminuer. Il n'y a pas encore de traitement spécifique pour ces patients; des essais cliniques de thérapie génique devraient débuter dans les années à venir [3, 4].

## Conclusion

La dystrophie maculaire de stargardt est une maladie héréditaire à transmission récessive, elle est bilatérale d installation progressive. Le diagnostic se base sur l'interrogatoire le fond d œil et les examens complémentaires (clichés auto fluorescents angiographie OCT et ERG).
